# Insights into the Relationships Between Herbicide Activities, Molecular Structure and Membrane Interaction of Cinnamon and Citronella Essential Oils Components

**DOI:** 10.3390/ijms20164007

**Published:** 2019-08-16

**Authors:** Laurence Lins, Simon Dal Maso, Berenice Foncoux, Anouar Kamili, Yoann Laurin, Manon Genva, M. Haissam Jijakli, Caroline De Clerck, Marie Laure Fauconnier, Magali Deleu

**Affiliations:** 1 Molecular Biophysics at Interfaces Laboratory, TERRA Research Centre, GX ABT, Université de Liège, 4000 Liège, Belgium; 2 Integrated and Urban Plant Pathology Laboratory, TERRA Research Centre GX ABT, Université de Liège, 4000 Liège, Belgium; 3 Laboratory of Chemistry of Natural Molecules, TERRA Research Centre, GX ABT, Université de Liège, 4000 Liège, Belgium

**Keywords:** Essential oils, plant plasma membrane, structure/activity relationships

## Abstract

Since the 50’s, the massive and “environmental naïve” use of synthetic chemistry has revolutionized the farming community facing the dramatic growth of demography. However, nowadays, the controversy grows regarding the long-term harmful effects of these products on human health and the environment. In this context, the use of essential oils (EOs) could be an alternative to chemical products and a better understanding of their mode of biological action for new and optimal applications is of importance. Indeed, if the biocidal effects of some EOs or their components have been at least partly elucidated at the molecular level, very little is currently known regarding their mechanism of action as herbicides at the molecular level. Here, we showed that cinnamon and Java citronella essential oils and some of their main components, i.e.,, cinnamaldehyde (CIN), citronellal (CitA), and citronellol (CitO) could act as efficient herbicides when spread on *A. thaliana* leaves. The individual EO molecules are small amphiphiles, allowing for them to cross the mesh of cell wall and directly interact with the plant plasma membrane (PPM), which is one of the potential cellular targets of EOs. Hence, we investigated and characterized their interaction with biomimetic PPM while using an integrative biophysical approach. If CitO and CitA, maintaining a similar chemical structure, are able to interact with the model membranes without permeabilizing effect, CIN belonging to the phenylpropanoid family, is not. We suggested that different mechanisms of action for the two types of molecules can occur: while the monoterpenes could disturb the lipid organization and/or domain formation, the phenylpropanoid CIN could interact with membrane receptors.

## 1. Introduction

Since the 50’s in industrial countries, the massive use of synthetic chemistry has revolutionized the farming industry regarding demographic growth. However, nowadays, the controversy rises about harmful effects of those products on human health and environment. Furthermore, conventional herbicides induce resistance that should be overcome by using bio-based products that targeted multiple and/or other molecular pathways than synthetic herbicides. In this context, the use of secondary metabolites from plants, such as essential oils (EOs), could be an alternative to chemical products [1,2]. In this view, a better understanding of their mode of biological action for new and optimal applications is of importance.

Herbicide effects of EOs, such as drastic growth decrease, severe chlorosis, or leave burning, have been described in the literature [3]. They were notably related to waxy cuticular layer removal, disruption of microtubule polymerization, cellular respiration decrease, mitosis inhibition, ion leakage and membrane depolarization, oxidative damages, or chlorophyll content decrease [4,5,6,7], but no detailed molecular mechanisms are published to our best knowledge.

Since EOs are composed of small amphiphilic molecules, they could cross the mesh of cell wall and directly interact with the plant plasma membrane (PPM). Modifying the lipid organization could lead to crucial cellular effects [8,9,10], notably on protein function [11,12]. The interaction of EOs with the cellular membrane is also responsible, at least partly, for their antimicrobial activities, which suggests that plasma membrane is one of the cellular targets of EOs. For bacteria, as well as for fungi, EOs were shown to affect the lipid membrane due to their lipophilic nature. They notably change the fluidity of the membrane by inducing perturbation of the membrane potential and/or permeability and fluidity and/or modifying the activity of ion channels (for a review, see [13,14]). Those effects can notably induce cell lysis or death by apoptosis or necrosis.

**Figure 1:**
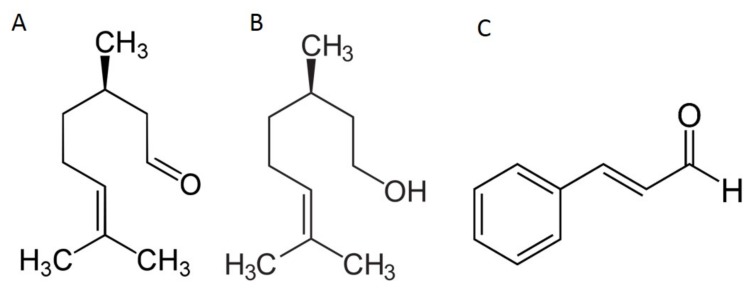
Structures of (+)-citronellal (CitA) (**A**); (+)-citronellol (CitO) (**B**); trans-cinnamaldehyde (CIN) (**C**).

In this context, we have investigated the herbicidal activities of two EOs, namely cinnamon and Java citronella and some of their main chemical components, namely trans-cinnamaldehyde (CIN), (+)-citronellal (CitA), and (+)-citronellol (CitO) in relation with their membrane activities. CitA and CitO are oxygenated monoterpernes, while CIN belongs to the aromatic phenylpropanoid family (Figure 1). The (+) enantiomer of CitA and CitO was chosen, because enantioselective effects were observed [15,16,17]. (+)-CitA was notably shown to be more active on microtubules on animal cells [15] (or plant cells [15]. This differential activity was also noticed for other monoterpenes, such as α-pinene [18].

CIN, CitA, and CitO have already been shown to affect biological membranes in their biocidal activities. For CitO, its antifungal activity was notably linked to the disruption of the cell membrane, followed by the leakage of cell constituents [19]. Cell disruption seems to be related to a modification of ergosterol content and production [20,21]. Lim and Shin [22] demonstrated that the effects of CitO on the membrane are not only due to the impairment of ergosterol biosynthesis, but also to a change in the lipid composition of the cell membrane. In human red cells, CitO was also shown to have an effect on sterol, by displacing membrane cholesterol [23].

As herbicide, CitO enhances solute leakage and induces ROS generation on root and shoot growth of *Triticum aestivum*. ROS production could result in lipid peroxidation and membrane damage [24].

Concerning CitA, several publications state that it has diverse biocidal activities in relation with the membrane. As antifungal, CitA can inhibit the growth of several *Aspergillus* species and of *Candida albicans* [25,26], notably by modifying the membrane fluidity and interfering with the membrane signalling proteins. CitA can also damage the cell membrane, reduce ergosterol levels by 50%, and lessen the plasma membrane ATPase activity by decreasing the glucose-induced H^+^ extrusion [27]. It was also shown that CitA inhibits the mycelial growth and spore germination of *Penicillium digitatum* by deteriorating the plasma membrane of the spores, leading to higher extracellular conductivity and the release of cell constituents [25].

As herbicide, CitA has been shown to inhibit weed emergence and early seedling growth, both in plant roots and shoots. It notably inhibits wheat seed germination [28]. In plants, its application leads to chlorosis and necrosis caused by a loss of chlorophyll and the reduction of cell respiration [29]. As an example, CitA was found to decrease the germination of *Digitaria horizontalis* and *Cenchrus echinatus* by 98%, and to diminish their chlorophyll and total protein content in cell by 80% and 90%, respectively [30]. CitA also triggered the disruption of cuticular wax, clogging of stomata, and rapid electrolyte leakage, as a consequence of the disruption of the membrane integrity [29]. Besides its activity on the membrane, CitA was also shown to affect the microtubule structure in plant cells [15,16]. CIN has been shown to have a broad spectrum of antibacterial activity, notably by affecting cell morphology, membrane integrity, and permeability, modifying the fatty acid composition [31,32,33,34]. By using lipid monolayers mimicking bacterial membrane, it was shown that CIN integrates the lipid layer, increases the fluidity, and alters the dipole moment [35]. However, the impact of CIN on the plasma membrane can differ. Mnif et al. [14] notably reported that CIN can inhibit the growth of *Escherichia coli* and *Salmonella typhimirium* without disintegrating the membrane or depleting intracellular ATP.

As an antifungal, CIN was shown to interact with the membrane of *Candida albicans* by reducing its ergosterol content by 55% [36]. It also increases the ROS production and impairs the cell membrane permeability and the cell wall integrity of *Penicillium italicum* [37].

As herbicide, CIN has only been shown to exert an inhibitory effect on seed germination, shoot and root growth of Chinese amaranth by 55%, 75%, and 85%, respectively [38].

To better understand the mode of action of those three small amphiphile molecules as herbicides, we have investigated and characterized their interaction with biomimetic PPM while using an integrative biophysical approach, since PPM could be one of the cellular targets of EOs.

## 2. Results

### 2.1. Herbicidal Effects of Cinnamon and Java Citronella Oils and Their Main Components

**Figure 2:**
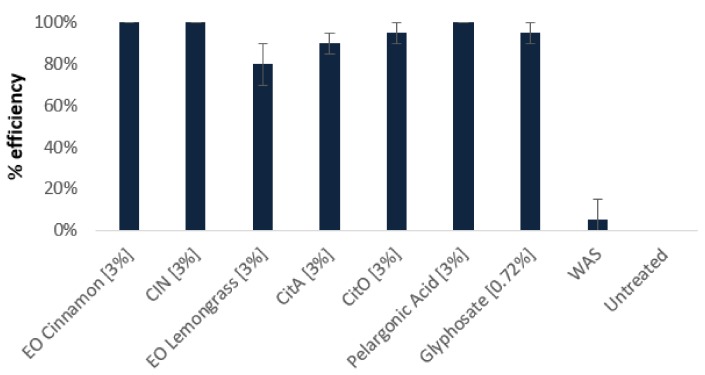
Herbicidal activity of cinnamon and Java citronella essential oils and their main components at 3%, CIN, CitA, and CitO respectively, after seven days. They are compared to glyphosate (0.72%) and pelargonic acid (3%) and to untreated plants or treated without active substance (WAS) (1% Tween 20 and 0.5% ethanol) – (*n* = 5).

Sixty and 57 different compounds were respectively identified in cinnamon and Java citronella Eos, revealing typical compositions for both studied oils [7]. The main component of the cinnamon EO is cinnamaldehyde (71.80%), followed by eugenol, caryophyllene, cinnamyle acetate, and linalool while Java citronella EO is mainly composed of citronellal (37.59%), geraniol (21.94%), citronellol (14.06%), limonene (5,63%), and eugenol (1,60%) (Figure S1). The other detected compounds are present in lesser amount. The total essential oils and CIN, CitO, and CitA were sprayed on *A. thaliana* leaves to evaluate their herbicidal activities. Those three molecules were chosen, since CIN is the main component of cinnamon and CitO and CitA have similar chemical structures (Figure 1). They were compared to glyphosate and pelargonic acid, which are the main active substances of commonly used herbicides. Figure 2 and Figure S2 show clearly that cinnamon and Java citronella EOs are as active as the conventional herbicides. The penetration tests revealed that Cinnamon EO, Java citronella EO, CIN, CitA, and CitO where all able to penetrate the leaves when applied on the surface. Indeed, in each experimental situation, the applied compound was found in the hexane extracted leaves that were rinsed by water, ethanol, and hexane, as described in Methods (data not shown) [39].

CIN, CitA, and CitO at 3% have almost the same activities as the whole oils seven days after the treatment (Figure 2). However, the EOs are more efficient than the molecules at lower concentrations (Figure S2). Regarding the kinetics of the EOs and their main components, they take more time than pelargonic acid to have their maximal efficiency (one day for PA and seven days for EOs). CIN and CitO at 3% have a quicker response than CitA at 3% (three days for CIN and CitO and seven days for CitA). At the lower concentration, CIN has a quicker response than CitA and CitO (three days for CIN and seven days for CitA and CitO-Figure S2). In addition to its herbicidal effect, we observed that CitA at 3% had a strong effect on the growth of the plants that were not completely killed (Figure S3, panel H).

The herbicidal effect that we observed could be linked to the fact that the plasma membrane of the plant cell could be one of the targets of the individual molecules from EOs, due to their lipophilicity and small size. To test this hypothesis, we performed in silico and in vitro biophysical assays to characterize the interaction of the individual molecules CIN, CitO and CitA with model membranes mimicking PPM. For this, we chose palmitoyl linoleyl phosphatidylcholine (PLPC), since phospholipids are one of the major components of plant plasma membranes (PPM), and PC is their most common phospholipid [40]. It is also reported that linoleic acid is one of the main fatty acids found in PPM phospholipids. Beta-sitosterol is a major sterol species in PPM [40], and it is also used in this study.

### 2.2. In Silico Prediction of The Insertion of CIN, CitO and CitA in Model Membranes

**Figure 3:**
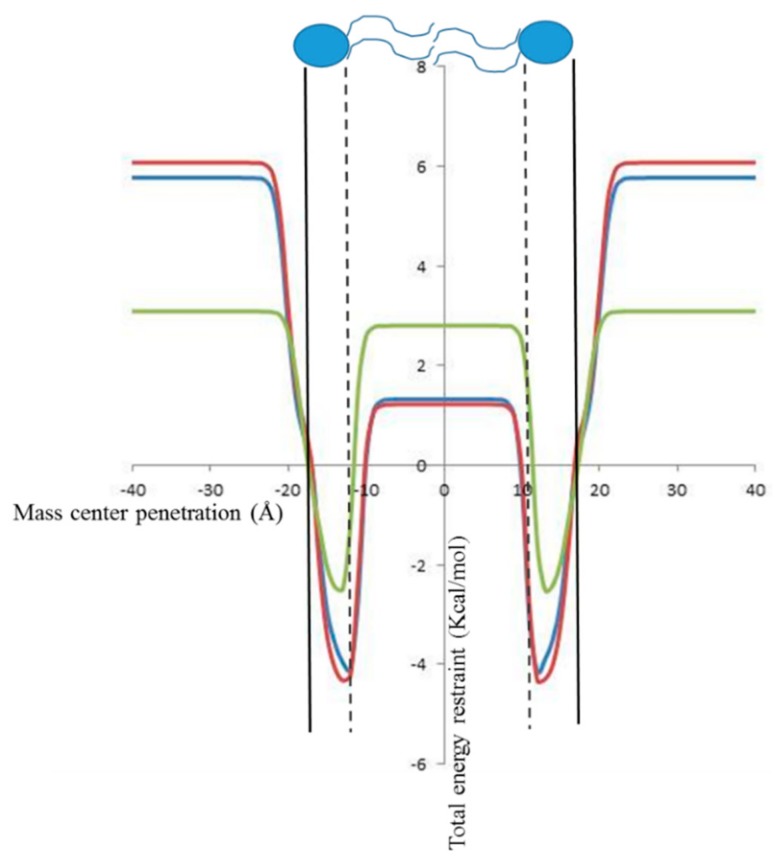
Evolution of the total restraint as a function of the molecule mass center penetration in an implicit bilayer obtained by IMPALA. The vertical black line corresponds to the interface between the lipid heads and the aqueous phase (*z* = 18 Å), the dotted line represents the interface between the lipid hydrocarbon chain and the hydrophilic head (*z* = 13.5 Å), and the center of the bilayer is at 0 Å. A cartoon of two lipid molecules (in blue) is presented to better visualize the different interfaces. Red curve: CitO, blue curve, CitA, green curve: CIN.

First of all, the membrane insertion capacity of the three molecules was tested while using the IMPALA procedure [41]. Briefly, this simple in silico method allows for predicting the insertion of a molecule into an implicit model bilayer. Figure 3 shows the evolution of the total restraint energy as a function of the penetration of the mass center of CIN, CitO, and CitA. For the three molecules, the total restraint energy is higher outside the bilayer than inside, which suggests that their insertion within the membrane is energetically favorable. Their most favorable position is near the interface between the lipid polar head and the alkyl tails. However, a difference is observed between CitO and CitA on one side and CIN. Indeed, the value of the restraint for CIN is almost the same in water (i.e.,, outside the membrane) as in the hydrophobic core of bilayer, while there is a significant difference for CitO and CitA that should favor their interaction with the hydrophobic part of the membrane as compared to a hydrophilic medium, such as water (Figure 3). This is in agreement with their respective octanol/water partition coefficient found on PubChem, 3.91 for CitO, 3.53 for CitA, and 1.90 for CIN.

**Figure 4:**
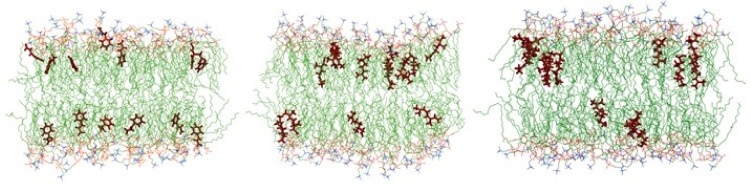
Snapshots after 100 ns of a 102 molecules palmitoyl linoleyl phosphatidylcholine (PLPC) and 26 molecules sitosterol bilayer with 13 CIN molecules (left panel), 13 CitA molecules (middle panel) and 13 CitO molecules (right panel). For the sake of clarity, water molecules are omitted. Dark red: herbicidal molecules, green: carbon atoms, red: oxygen atoms, orange: phosphor atoms, blue: nitrogen atoms.

We performed molecular dynamics (MD) simulations on a membrane made of 102 molecules of PLPC and 26 β-sitosterol to have a more realistic model. Thirteen molecules of either CIN, CitO, or CitA were put in the water medium and the evolution of the system was followed during 100 ns. Figure 4 displays the snapshots at the end of 100 ns simulations, with 13 molecules of each molecule tested being inserted into the model membrane.

**Figure 5:**
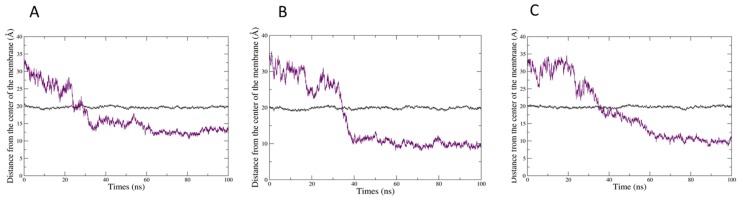
Evolution of the mean distance (in Angstroms) between the centre of the bilayer and the phosphate groups of PLPC (dark grey curve) and that of (**A**) 13 molecules of CIN, (**B**) 13 molecules of CitA, and (**C**) 13 molecules of CitO (purple curve).

The three molecules are able to penetrate into the bilayer, CitO and CitA appearing to be more embedded into the membrane. This is confirmed by the evolution of the mean distance of the polar head of PLPC as compared to that of the mass center of the herbicidal molecules (Figure 5). Hence, the CIN mass centre is around 12–13 angstroms from the centre of the bilayer, while CitO and CitA are around 10 angstroms.

**Figure 6:**
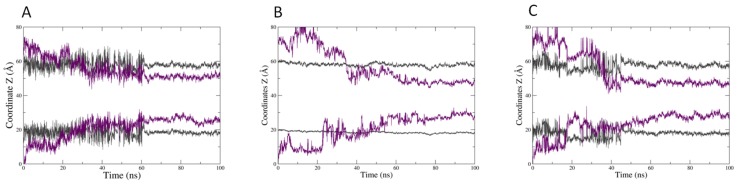
Transversal cut of the lipid bilayer in the presence of (**A**) 13 molecules of CIN (**B**) 13 molecules of CitA and (**C**) 13 CitO molecules. Dark grey: mean z coordinates of the mass centre of the phosphate atoms of PLPC molecules, purple: mean z coordinates of the mass centre of the herbicidal molecules.

A different behaviour is also noticed when looking at the evolution of the mean Z coordinates of the both the mass centre of each EO component and that of the lipid phosphate groups, which represents a transversal cut of the system (Figure 6). If CitA (Figure 6B) can stably penetrate the bilayer after 40 ns, CIN appears to have a less stable and shallower membrane penetration. Fluctuations in the position of both CIN and the phosphate group of PLPC are observed, during 60 ns of the simulation time (Figure 6A). This indicates that the interaction of CIN with the membrane surface is less stable, the molecules appear to go in and then out of the membrane, which induces a perturbation of the lipid polar heads. CitO stably and deeply inserts after 40 ns as for CitA and it seems to have an intermediate effect on the position of the lipid phosphate groups (Figure 6C). Hence, the deeper insertion of CitA and CitO as compared to CIN is confirmed.

### 2.3. In Vitro Biophysical Assays

In silico approaches suggest that a different interaction behaviour should be observed between CitO and CitA on the one hand and CIN on the other. The ITC experiments were then carried out to study the experimental ability of each EO main component to partition into lipid bilayers and to thermodynamically characterize the interactions that could occur.

Typical raw data of an ITC experiment for CitO, CitA, and CIN are shown in Figure S4. ITC raw data for CitA and CitO display a gradual decrease of the positive heat flow signal over the course of the successive injections, which is characteristic of a binding event, while it is not the case for CIN, which indicates no significant interaction with the liposomes. The thermodynamic parameters (Table 1) that were calculated from the fitting curves indicate that the binding of CitA or CitO is spontaneous (∆G < 0), endothermic (∆H > 0), and leads to a positive change of entropy (∆S > 0), even if their affinity for the PLPC/sitosterol bilayer is not very high (K values are comprised between 0.01 and 0.03 mM^−1^).

The absolute value of entropy change is higher than the one of enthalpy change, which indicated that the binding is entropy driven. This is probably due to the transfer of the hydrophobic chain of CitA or CitO from the water to the hydrophobic core of the liposomes.

This is supported by the fact that there is no obvious difference between the thermodynamic parameters of CitA and CitO, having the same hydrophobic tail.

We performed permeability assays using liposomes having the same composition as for ITC experiments to see whether the interaction with the model membrane induces a perturbation of the lipid organization. Figure S5 shows that the interaction does not give any destabilization of the bilayer as the HPTS fluorescence values are below 2%, even at molar ratio of 1/1 (EO/lipid).

Adsorption experiments into PLPC, β-sitosterol, and PLPC/sitosterol monolayers were performed to investigate the potential lipid specificity in the interaction and to highlight a possible difference in the mechanism of interaction between the molecules.

CitA and CitO, but not CIN, are able to adsorb into PLPC, β-sitosterol, or PLPC/sitosterol monolayer (Figure S6). The equilibrium state is reached faster in the case of β-sitosterol.

**Figure 7:**
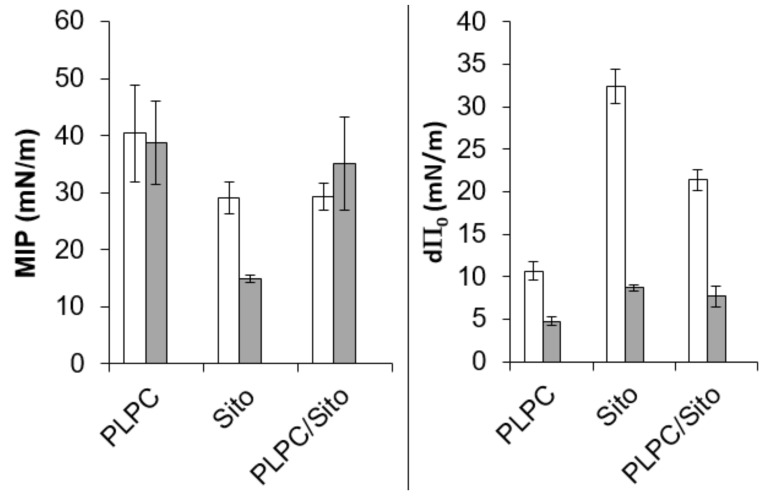
Adsorption of CitA (white) and CitO (grey) into lipid monolayers: PLPC, β-sitosterol or PLPC/β-sitosterol. (**A**) Maximal insertion pressure (MIP) and (**B**) differential Π_0_ (d Π_0_) values. For CIN, MIP, and d Π_0_ were not quantifiable.

The binding parameters that were obtained from the plots (Figure S7) of the surface pressure variation at the equilibrium versus the initial surface pressure obtained for the single lipid monolayer and the binary lipid composition are shown in Figure 7.

In the case of CitA and CitO, and for the binary lipid composition, the maximal insertion pressures (MIP) that are linked to the penetration power of EO main components are close or higher than the lateral pressure supposed to prevail in natural membranes (30–35 mN/m) [42]. It suggests that CitA and CitO, but not CIN, can be inserted into natural PPM. The positive values of d P_0_ indicate that the PPM lipids have an attractive effect on CitA and CitO. This effect is higher for CitA than CitO.

Individual lipid monolayers, and especially that of β-sitosterol, show also a higher attractive effect on CitA than on CitO. However, in terms of penetration power (MIP), PLPC seems to be more favorable to the penetration and stabilization of both molecules. The physical state of the monolayer that is more rigid in the presence of β-sitosterol can have an influence on the insertion behavior of the two molecules. It can be further noted that CitO has peculiar adsorption behavior. Indeed, there is a rapid and important increase of the surface pressure just after the injection of CitO, followed by a rapid decrease, not observed (or not significant) for CitA (Figure S6). One assumption is that CitO rapidly evaporates after injection, inducing this “increase/decrease” phenomenon. However, in the case of β-sitosterol, the decrease is less rapid (Figure S6B), which suggests that another phenomenon could occur, such as removal of lipid molecules from the monolayer to the subphase. This hypothesis is further supported by the fact that, at a higher initial surface pressure of β-sitosterol (above 15mN/m), there is a decrease of the surface pressure with time, to values that are lower than the initial pressure (Figures S7B and S8).

## 3. Discussion

In this paper, we showed that cinnamon and Java citronella oils and their main respective components (i.e.,, CIN, CitO, and CitA) have herbicide effects on the model dicotyledon plant, *A. thaliana* and are as potent as the active substance of commercial herbicides, namely glyphosate and pelargonic acid. The three molecules have already been studied for their herbicide potential. CitO and CitA were shown to inhibit germination, root, and/or shoot growth of different plants [24,28,29,30]. On the other hand, CIN was only reported to be phytotoxic on Chinese amaranth [38]. The mechanisms that are involved in the toxicity are supposed to affect either the energy metabolism, such as photosynthesis and/or involve ROS production. Besides, effects on microtubules have been observed [15,16]. Electrolyte leakage is often observed, which indicates that the integrity of the cell membrane is affected. These studies suggest that the cell membrane could be a target through which phytotoxicity is exerted.

The plasma membrane is also one of the action sites described for the antimicrobial activities of EOS, so we tested whether the individual compounds of the two EOs selected were able to interact with a model membrane that mimics PPM. Our complementary in silico and in vitro biophysical approaches indicated that CitO and CitA can stably interact with plant lipids, while CIN has no stable interaction with the membrane.

For CIN, MD approaches suggested that it could not stably interact with the model membrane over 100ns, but can, however, penetrate at the level of the lipid polar heads and disturb them. This was not experimentally observed, since no interaction could be noticed in ITC or Langmuir monolayer assays. It should be underlined that the timescale of the MD simulations (nanoseconds) is far from the in vitro timescale (seconds to minutes). Hundreds of nanoseconds might not be sufficient to observe CIN molecules to get out of the membrane. Nevertheless, we cannot completely rule out that the volatile nature of CIN could also be involved in the absence of interaction with the membrane. In the literature, CIN has been described as interacting with the monolayers of lipids mimicking bacterial membrane [35]. The different results might be explained by the fact that CIN could have an affinity for bacterial lipids, and not for plant lipids that are quite different in their physico-chemical properties.

On the other hand, CitO and CitA have comparable affinities for plant lipids, since the thermodynamic parameters for their interaction with PLPC/sitosterol liposomes are similar. The interaction is entropy-driven, due to the fact that their alkyl chain can interact with the lipid hydrocarbon chains, as observed in the MD simulations. When looking at the effects of the individual lipids, we observed that PLPC has an attractive effect for both molecules, especially for CitA. Sitosterol has an even more pronounced effect on the latter. However, we noticed a peculiar behavior of CitO in the presence of sitosterol monolayer. There is a rapid increase of surface pressure in the seconds after the injection of CitO, which is followed by a gradual decrease of the pressure. If the evaporation of CitO could be responsible for this observation, another hypothesis can be put forward: CitO could be able to remove sterol molecules from the lipidic film. This is supported by the fact that CitO is able to displace cholesterol molecules from its phospholipid partners [23]. This effect is referred to as cholesterol activation. We can assume that CitO could have the same effect on sitosterol, presenting a similar structure as cholesterol. The differences that were observed between CitO and CitA at the level of their interaction with the sterol could also be related to the different kinetics and concentration effects that were observed in the *in planta* assays (Figure 2 and Figure S3). Here, we have to mention that conversion of CitA to CitO *in planta* is also possible, since Dudai et al. have observed that CitA can be bioconverted into CitO in wheat seeds [17]. In the same way, citral was shown to be converted to geraniol and nerol and limonene to carvacrol *in planta* [16]. Analysis of the hexane extracts obtained from rinsed leaves (penetration tests) showed that no significant conversion can be observed within the tested frame of time (2 h). for example, less than 1 % of CitA applied as pure compound was converted into CitO two hours after application (data not shown) [38].

If CitO and CitA were shown to interact with model PPM, they do not exert their phytotoxic effect by simply destroying the membrane. Indeed, leakage assays clearly indicated that no perturbation of the membrane is observed. This is in agreement with the fact that the antimicrobial effects of some terpenes, such as γ-terpinene or p-cymene, are not necessarily linked to significant membrane perturbation [43]. The latter should be dependent on the lipidic composition and net surface charge [43]. Subtler mechanisms for membrane perturbations, such as the modification of lipid nano- or microdomains, which are involved in signaling processes, could also be involved in the toxic effect observed *in planta*. This was already suggested for other natural molecules such as surfactin [44]. The latter is a bacterial amphiphile molecule that is able to elicit the plant defenses by acting on the lipid part of the membrane.

Other molecular mechanisms can be assumed for CIN. This molecule, as well as molecules belonging to the phenylpropanoid family, such as eugenol, were shown to be agonists and ligands of mammal membrane ion channels, namely transient receptor potential Ankyrin 1 (TRPA1) and transient receptor potential vanilloid 1 (TRPV1) [45,46,47]. A yet-to-discover plant membrane protein could be involved in the toxic effects of CIN on plants.

## 4. Materials and Methods

### 4.1. Chemicals

1-palmitoyl-2-linoleoyl-sn-glycero-3-phosphocholine (PLPC) and β-sitosterol were purchased from Avanti Polar Lipids, Inc. (Alabaster, Alabama, USA). The essential oils of *Cinnamomum zeylanicum* Blume (cinnamon) and *Cymbopogon winterianus* Jowitt (Java citronella) were from Pranarom (Belgium). All other chemicals were purchased from Sigma Aldrich Inc. (Saint Louis, Missouri, USA). The enantiomers of the main EO components were trans-cinnamaldehyde (CIN), (+)-citronellal (CitA), and (+)-citronellol (CitO).

### 4.2. Chromatographic Analysis of Cinnamon and Java Citronella Essential Oils

Ten milligrams of essential oil were dissolved in 100 mL of hexane and analyzed by GC-MS for identification and GC-FID for quantification. For each essential oil, the analysis was performed in triplicate according to Tanoh et al. [48], with slight modifications. GC-MS was carried out while using an Agilent GC system 7890B (Agilent, Santa Clara, CA, USA) fitted with a split-splitless injector and coupled to an Agilent MSD 5977B detector. One microliter of 0.01% essential oil solution was injected, and the analytical conditions were fixed, as follows: injection mode: splitless at 300 °C; VF-WAXms capillary column (Agilent, Santa Clara, CA, USA) (30 m × 0.25 mm, df = 0.25 μm); temperature program: from 40 °C (5 min.) to 225 °C at a rate of 2 °C/min. The carrier gas was helium at a flow rate of 1.6 mL/min. The mass spectra were recorded in Electron Ionization mode at 70 eV (scanned mass range: 40–400 m/z). The source and quadrupole temperatures were fixed at 230 °C and 150 °C, respectively. The component identification was performed on the basis of chromatographic retention indices (RI) and by comparison of the recorded spectra with a computed data library (Pal 600K®). RI values were measured on a VF-WAXms column (Agilent, Santa Clara, CA, USA). RI calculations were performed in temperature program mode according to [49,50]; a mixture of homologues n-alkanes (C7–C30) was used under the same chromatographic conditions. The main components were confirmed by comparison of their retention and MS spectrum data with co-injected pure references (Sigma, Darmstadt, Germany). The quantification of the main EO’s components was performed by analyzing the EOs in exactly the same conditions by GC-FID while using individual calibration curves that were prepared with pure standards (Sigma, Darmstadt, Germany). The quantification was performed in triplicate for each EO.

### 4.3. Penetration and Stability of Essential Oils and Pure Compounds Tests

To verify that, EO emulsions, CitA, CitO, and CIN effectively penetrate the leaves and evaluate whether any biological conversion occurs during the test period, the following experimentation was undertaken, adapted from Lichiheb et al. [51].

Five fully expanded leaves of *A. thaliana* were detached, placed in glass Petri dishes. Six 10 µL droplets of the tested compound (EOs, EO emulsions, CIN, CitA, CitO) were dropped on the adaxial face of each side of the main vein of the leaves and allowed to stand for 2 h at room temperature. Each leaf was successively rinsed five times by 1 mL of water, ethanol, and hexane. The rinsed leaves were then grinded in liquid nitrogen and immediately extracted by sonication with 5 mL of hexane containing 100 µL of a 5 mg/mL benzylic acid solution. The supernatant (0.5 µL) was injected in the GC-MS and analysed, as described above (*n* = 5).

### 4.4. Herbicide Tests on A. Thaliana

From a practical point of view, herbicides are used in aqueous solution. An emulsion of EOs in water was necessary in order to obtain a formulation that was as stable as possible. To do so, Tween 20 (1%) and ethanol (0.5%) were used.

The EOs of *C. zeylanicym* and *C. winterianus* (each at a concentration of 3%), as well as CIN (2.15% and 3%), CitA (1.13% and 3%), and CitO (0.03% and 3%), were tested. The tested concentrations for individual molecules were chosen to be representative of 1: the proportion of their abundance in the whole EOs, and 2: the concentration used to test the whole EOs. Biological assays were made on *Arabidopsis thaliana* (L.) Heynh. The plants were treated at the “two-leaves” stage. Pelargonic acid (PA) (3%) from Compo® and glyphosate (0.72%) from Monsanto® were used as positive controls as well as a formulation without active substance (WAS), only containing Tween 20 (1%) and ethanol (0.5%).

For glyphosate and PA, the pulverisation was made following the recommendation use (30 m^2^/l for glyphosate and 10 m^2^/l for PA). For the EOs and individual molecules formulations, the pulverisation was made following the PA recommendation use (10 m^2^/l). Five repetitions (pots containing 20 seeds) were made for each object.

After spraying, the plants were placed in a greenhouse and the number of completely dead plants was determined each day during seven days. The herbicidal effect was determined by comparing the number of completely dead plants seven days after the treatment to the initial number of plants in each pot.

### 4.5. In Silico Approaches to Study the Interaction of the EOs Main Components with Lipids

The three-dimensional (3D) structures of CIN, CitA and CitO, PLPC, and β-sitosterol were constructed while using HyperChem software (Hyperchem 7.1, Hypercube Inc., Gainesville, FL, USA). The molecular geometry was optimized with the steepest-descent method while using the MM+ force field, and a systematic analysis of the torsion angles using the structure tree method was performed, as described previously [52]. The most probable structure corresponding to the lowest conformational energy was used for further calculations.

The insertion of the molecule into an implicit bilayer was computed by the IMPALA procedure, as described in Ducarme et al. [41]. Briefly, an implicit membrane is described as a continuous medium whose properties vary along the axis perpendicular to the bilayer plane (z axis). The membrane properties are represented by energy restraints. The EO molecule is systematically moved along the z axis by 1 Å steps, from one side of the membrane to the other and the restraints are calculated for each position. A profile of the energy restraints as a function of the penetration into the implicit bilayer is obtained.

### 4.6. Molecular Dynamics Simulations

Simulations have been performed with GROMACS 5.0.2 and the united atom GROMOS 53a6 force field [53]. The topologies of β-sitosterol, CIN, CitO, and CitA were obtained with Automatic Topology Builder [54]. A PLP [55] C topology that was derived from Berger Lipids forcefield [56] and developed by Peter Tieleman’s group [57] was used. It is available for download on its website. Bilayers containing 102 PLPC molecules and 26 β-sitosterol molecules were generated and hydrated by using Memgen [58]. The system was solvated with SPC water [59]. The membrane firstly underwent an energy minimization step, followed by a 100 ps NVT equilibration and by a 1 ns NPT equilibration. A production run of 500 ns of the membrane was realized to stabilize the membrane. After the stabilization of the membrane, thirteen herbicide molecules (lipid: herbicide molar ratio of 10:1) distant of 1nm from each other and from the limit of the water box were inserted. The molecules were placed on one side of the membrane at 0.8 nm from the PLPC phosphate. The system then underwent a 100 ps NVT equilibration, followed by a 100 ps NPT equilibration, during which the herbicides were under position restraints and then 100 ns production runs were performed. For the production runs, temperature was maintained to an average value of 298K by using the Nose–Hoover thermostat [60,61] with a τ_T_ = 0.5 ps. Semi-isotropic pressure (1 bar) was maintained by using the Parrinello-Rahman [55] barostat with a compressibility of 4.5 ×10^−5^ bar^−1^ and τ_P_ = 2 ps. Electrostatic interactions were treated by using the particle mesh Ewald (PME) method [62]. A cut-off of 1nm was used for Van der Waals interactions. Bond lengths were maintained with the LINCS algorithm The trajectories were analyzed with GROMACS tools, as well as with homemade scripts and they were visually analyzed with VMD [63] and PYMOL (The PyMOL Molecular Graphics System) software packages.

### 4.7. In Vitro Biophysical Assays to Study the Interaction of CIN, CitO and CitA with Model PPM

#### 4.7.1. Liposome Preparation

Large unilamellar vesicles (LUVs) were formed for isothermal titration calorimetry (ITC). Small amounts of lipids (PLPC/sitosterol 80/20 molar ratio) were dissolved into chloroform-methanol (2:1) in a round-bottom flask. A rotary evaporator was used to remove solvent under low pressure and the flask was then kept overnight under vacuum to remove the solvent traces. The lipid film was then hydrated with 10 mM TRIS - HCl buffer at pH 7 prepared from Milli-Q water. The flask was maintained at a temperature (~ 40 °C) well above the transition phase temperature of the lipid for at least 1 h and then vortexed for 1–2 min. every 10 min. to form multilamellar lipid vesicles (MLVs). Thereafter, the MLV suspension underwent five freeze-thaw cycles. In order to get LUVs, MLV suspension was then extruded 15 times through polycarbonate filters with a pore diameter of 100 nm.

#### 4.7.2. Isothermal Titration Calorimetry

The ITC measurements were performed with a VP-ITC from Microcal (Microcal Inc., Northampton, MA, USA). The sample cell contained 1.4565 mL of a solution of CIN, CitA, or CitO (132 µM) that was dispersed from a DMSO high concentrated stock solution into the same buffer as the LUV suspension. The reference cell was filled with mQ water. Small aliquots of LUV suspension were added to the sample cell with a software-controlled syringe. The first injection was 2 µL and was not taken into account for data treatment. It was followed by 28 successive additions of 10 µL, with an interval of 600 s. A LUV concentration of 5 mM was used for the three molecules. 

The data were processed by software ORIGIN 7 (Originlab, Northampton, MA, USA) while using the cumulative model, as described in Razafindralambo et al. [64].

#### 4.7.3. Adsorption Experiments into a Lipid Monolayer

Adsorption experiments were performed in a KSV Minitrough (Helsinki, Finland, 7.5 × 20 cm^2^). The subphase was Tris-HCl buffer pH 7 prepared from Milli-Q water (∼80 mL) with a constant temperature at 22.0 ± 1.0 °C. The subphase was continuously stirred with a magnetic stirrer. Pure PLPC or sitosterol molecules or a mix of the two lipids (PLPC/sitosterol 80/20 molar ratio) in chloroform/methanol (2/1 v/v) solvent, was spread at the air−water interface to reach the desired initial surface pressure. After 15 min. of waiting for solvent evaporation and film stabilization, CitO, CitA or CIN in DMSO solution was injected underneath the preformed lipid monolayer. The final subphase concentration was 67.5 μM. Their adsorption to the lipid monolayers was followed by the increase in surface pressure. As a control experiment, the same volume of pure DMSO was injected underneath the lipid monolayer, and no change in the surface pressure was observed. Maximal Insertion Pressure (MIP) corresponds to the surface pressure beyond which no absorption can happen. It was obtained by linear regression of the plot ΔΠ vs Πi at the intersection with the *x* axis. The “differential Π_0_ (dΠ_0_)” corresponds to the difference between ΔΠ_0_ which is the y-intercept of the linear regression of the ΔΠ vs Πi plot, and Πe which is the surface pressure increase at the equilibrium obtained in an independent experiment performed at the same CitA, CitO or CIN concentration but without lipids spread at the interface. A positive dΠ_0_ means an attractive effect of the tested lipid on the molecule adsorption. A negative value of dΠ_0_ points out the unfavorable impact of lipid on the molecule insertion. The uncertainties in MIP and the ΔΠ_0_ were calculated as described previously [65].

#### 4.7.4. Permeability assays

Membrane permeabilization was followed as described in Van Bambeke et al. [66]. Release of 8-hydroxypyrene-1,3,6 trisulfonic acid (HPTS) co-entrapped with and quenched by p-xylene-bis-pyridinium bromide (DPX) from LUVs can be monitored by the fluorescence increase upon dilution following their leakage from the vesicles. The dried lipid films were hydrated with a solution of HPTS and DPX in a 40 mM glycine-NaOH mixture adjusted to pH 11 at a concentration of 31.8 and 35 mM, respectively. After formation of LUVs, the unencapsulated dye was eliminated by passage on a sephadex G75 column. The LUVs were diluted to a final lipid concentration of 50 µM in Tris 10 mM, buffer pH 7. CitA, CitO or CIN was added and fluorescence intensities were immediately recorded. 

The percentage of HPTS released was defined as:

[(Ft − Fcontr)/(Ftot − Fcontr)] × 100

where Ft is the fluorescence signal measured after 15 min. in the presence of CitA, CitO or CIN, Fcontr is the fluorescence signal measured at the same time for control LUVs, and Ftot is the total fluorescence signal that is obtained after the complete disruption of the LUVs by 0.05% Triton X-100. All of the fluorescence determinations were performed at room temperature on a Perkin Elmer LS-50B Fluorescence Spectrophotometer (Perkin-Elmer Ltd., Norwalk, CT, USA) while using λ exc of 450 nm and a λ em of 512 nm.

## 5. Conclusions

In conclusion, CitO, CitA, and CIN are efficient herbicide molecules when spread on leaves. The PPM could be one site of action for CitO and CitA, but not for CIN, which could be related to their different chemical structure. The membrane activity of the formers is not leakage, but probably a more subtle effect on membrane domains or on membrane properties.

Further studies on the effects of CitO and CitA on membrane micro/nanodomains and on the physical state of the lipids should be carried out. An important class of plant lipids has not been investigated in this study, namely GIPC sphingolipids [67]. However, they are not commercially available for the moment. Another important issue for a better understanding of the toxic effects of EO compounds at the molecular level is to study their effects on the plant gene expression and metabolic pathways. This is currently under investigation in our group.

At the level of the effects on plants, the study of synergistic/antagonistic herbicidal effects between the different components of essential oils is also currently studied, as well as the penetration kinetics of these compounds into the different tissues of the leaf, together with the development of formulations, allowing for slow and controlled release [68]. All of those aspects should help to better understand the effects of the individual components of cinnamon and Java citronella EOs at the molecular level. This should lead to an optimal formulation of a natural herbicide targeting multiple and/or other molecular pathways as compared to conventional herbicides.

## Figures and Tables

**Table 1:** Thermodynamic parameters characterizing the interactions of the main chemical EO components with PLPC/sitosterol large unilamellar vesicles (LUVs).

	K (mM^−1^)	∆H_D_^w^ ^→b^ (kJ·mol^−1^)	T∆S_D_^w^ ^→b^ (kJ·mol^−1^)	∆G_D_^w^ ^→b^ (kJ·mol^−1^)
**CitA**	0.03 ± 0.00	5.1 ± 0.6	21.6 ± 0.3	−16.5 ± 0.9
**CitO**	0.01 ± 0.01	5.1 ± 0.5	23.3 ± 0.5	−18.5 ± 0.5
**CIN**	Not quantifiable	Not quantifiable	Not quantifiable	Not quantifiable

## Supplementary Materials

Supplementary materials can be found at https://www.mdpi.com/1422-0067/20/16/4007/s1.
