# Supplementary material for: Insights into the Relationships Between Herbicide Activities, Molecular Structure and Membrane Interaction of Cinnamon and Citronella Essential Oils Components
Source: Int J Mol Sci. 2019 Aug 16;20(16):4007. doi: 10.3390/ijms20164007 (PMC6720526; doi:10.3390/ijms20164007)
Supplement: Supplementary file 1 [file ijms-20-04007-s001.pdf]

Supplementary Materials: Supplementary materials can be found at www.mdpi.com/xxx/s1.

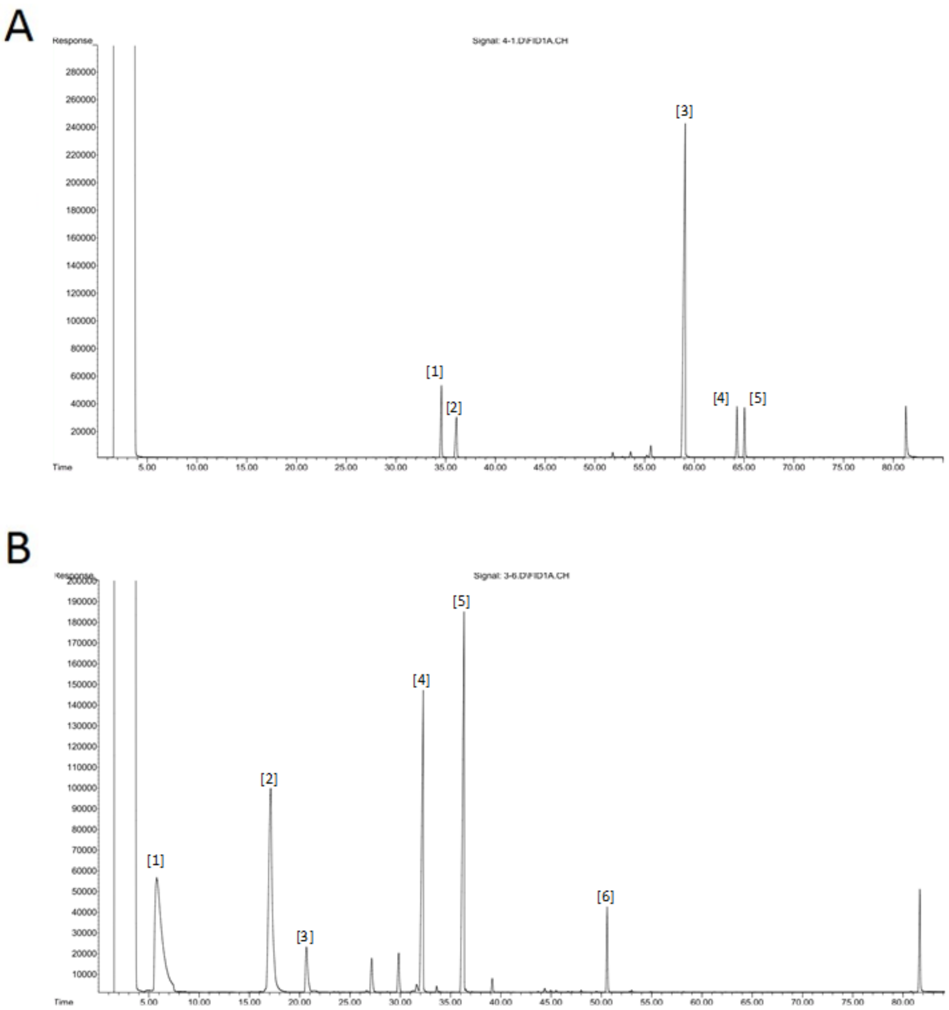

Figure S1. A. Chromatographic analysis of Cinnamon EO – [1] Caryophyllene, [2] Linalool, [3] Cinnamaldehyde, [4] Cinnamyl acetat, [5] Eugénol; **B**. Chromatographic analysis of Java citronella EO – [1] Limonene, [2] Citronellal, [3] Linalool, [4] Citronellol, [5] Géraniol, [6] Eugénol.

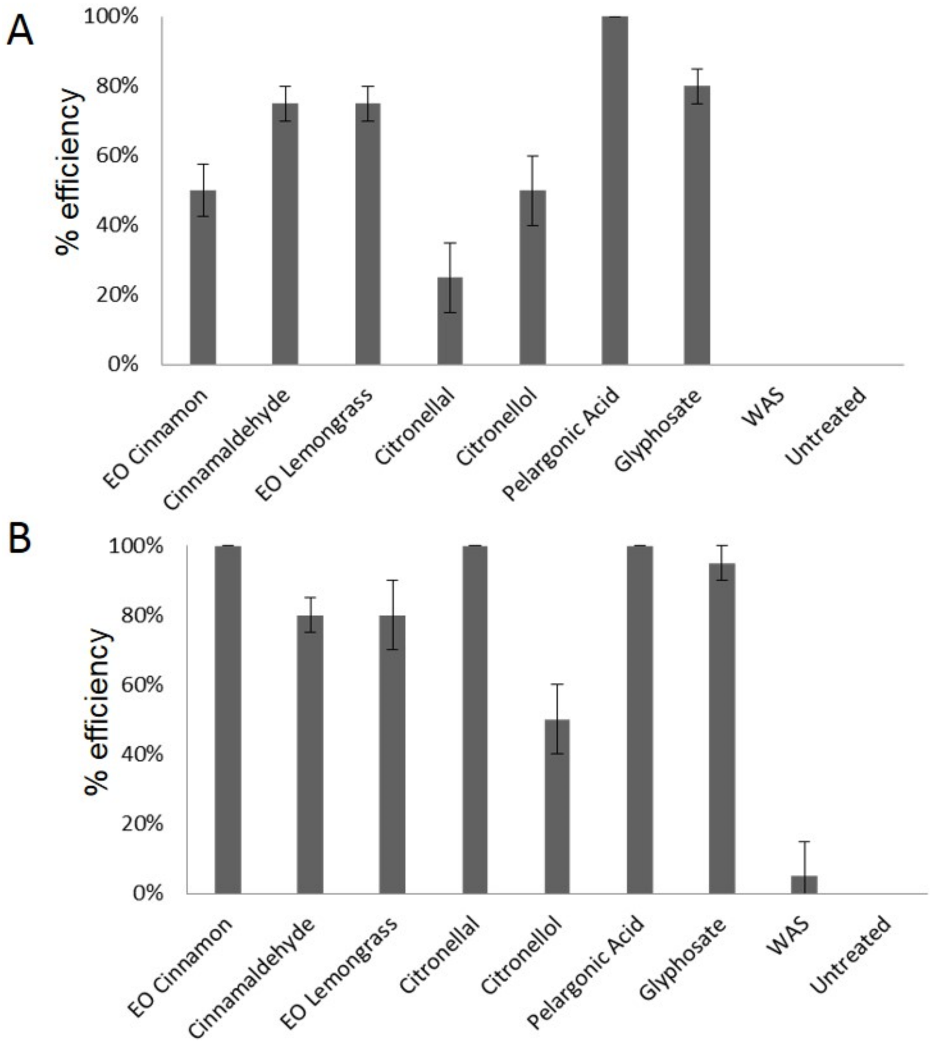

Figure S2. Herbicidal activity of cinnamon and Java citronella essential oils (3%) and their main components, CIN (2.15%), CitA (1.13%) and CitO (0.03%) respectively after 3 (**A**) and 7 (**B**) days. They are compared to glyphosate (0.72%) and pelargonic acid (3%) and to untreated plants or treated without active substance (WAS) (1% Tween 20 and 0.5 % ethanol) – (*n* = 5).

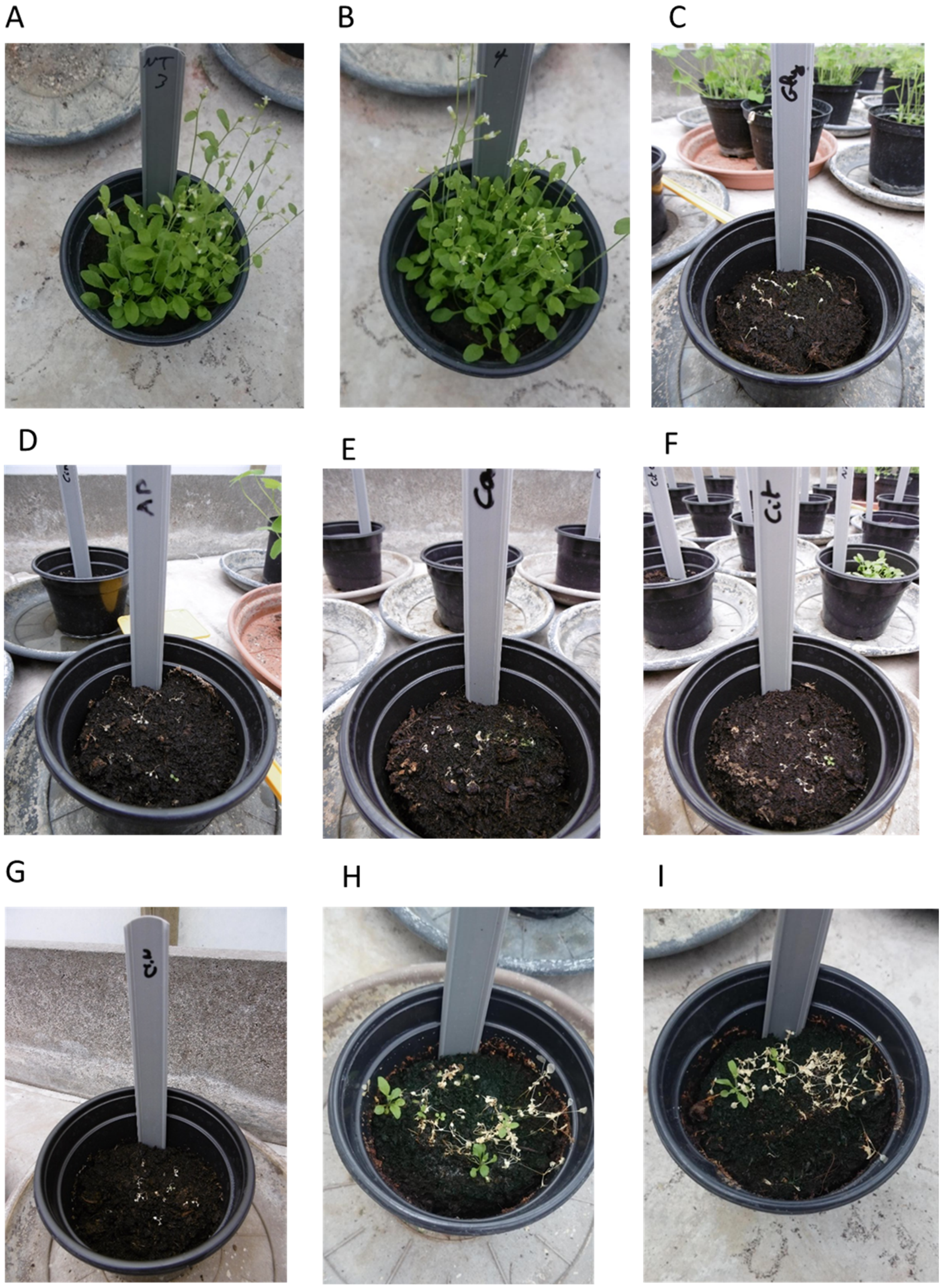

Figure 3. pictures of the application of the different products tested on *A. thaliana* leaves after seven days. **A**. Non treated plants; **B**. treated with no active substance; **C**. treated with glyphosate; **D**. treated with pelargonic acid; **E**. treated with cinnamon oil [3%]; **F**. treated with Java citronella oil [3%]; **G**. Treated with CIN [3%]; **H**. treated with CitA [3%]; **I**. treated with CitO [3%].

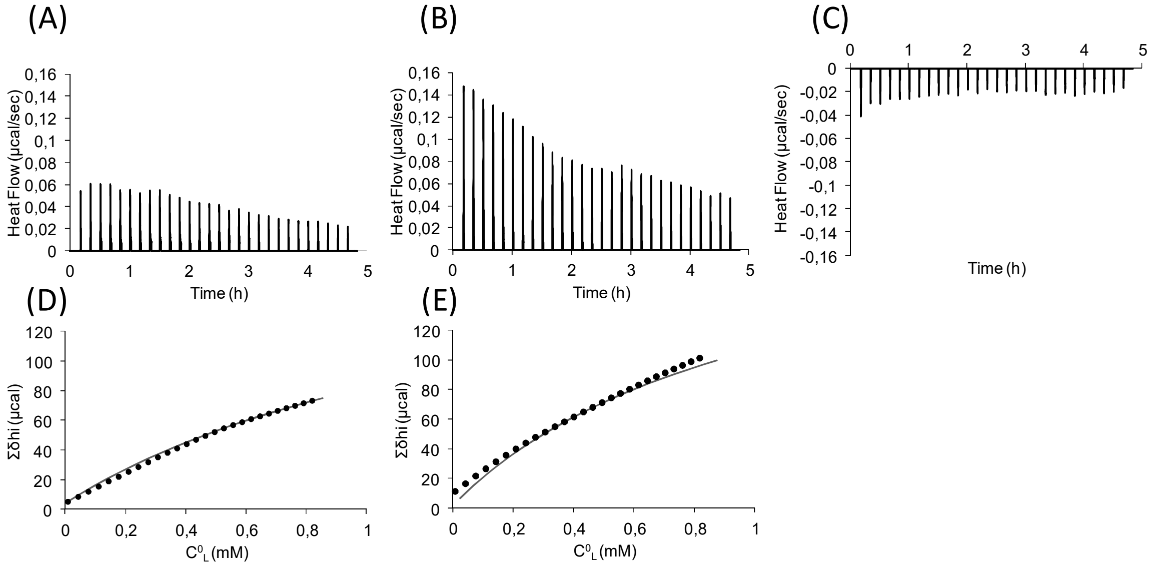

Figure S4. raw data from ITC experiments. Each peak corresponds to a single injection of 10µL of PLPC/sitosterol LUV suspension at 5 mM into a solution at 132 µM of **(A)** CitA, **(B)** CitO and **(C)** CIN at 26 °C. LUV suspensions and EO individual molecule solution were buffered at pH 7.4 with Tris-HCl. Lower panel: cumulative heats of binding (Σδhi) as a function of lipid concentration in the measure cell (C^0_L) (**D**) CitA and (**E**) CitO. No fitting has been carried out with CIN due to an absence of interaction.

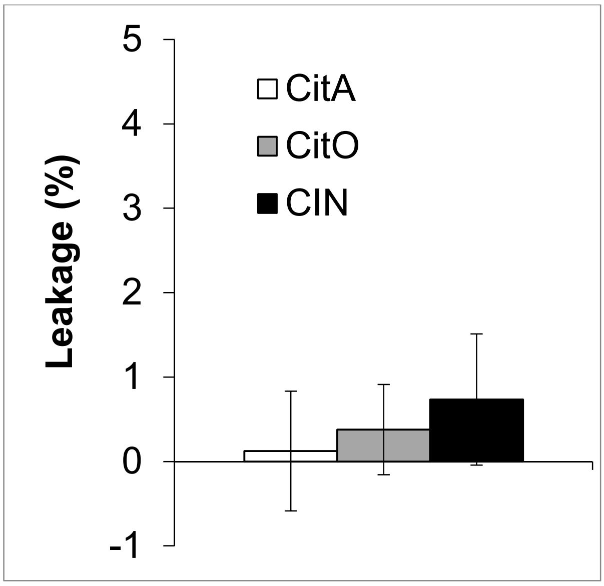

Figure S5. Mean relative HPTS release from PLPC/sitosterol (80/20) LUVs in the presence of CIN (black), CitA (white) or CitO (grey) for a molecule/lipid molar ratio of 1:1 after 15 minutes (the same ratio as obtained in the ITC experiment at the 4th injection). 100% is obtained after addition of Triton X100.

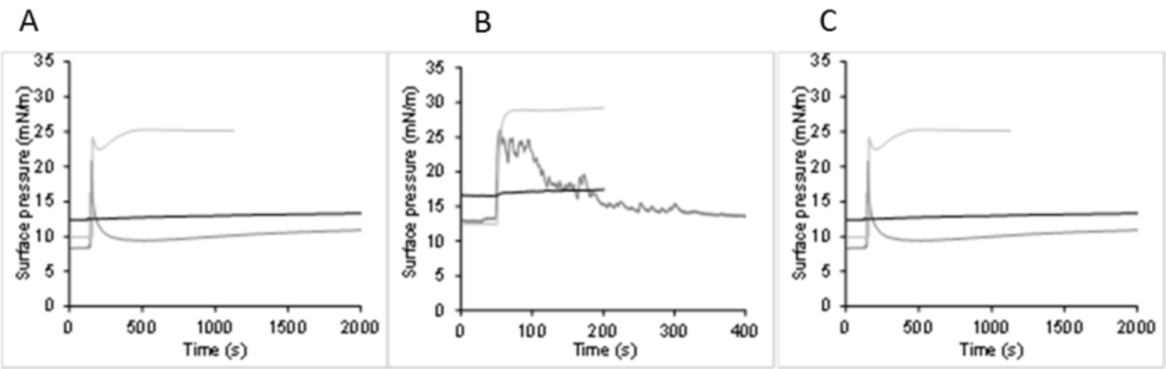

Figure S6. Adsorption of CitA (light grey), CitO (dark grey) and CIN (black) into a. (**A**) PLPC (**B**) sitosterol or (**C**) PLPC/sitosterol monolayer. Evolution of the surface pressure as a function of time for an initial surface pressure of the lipid monolayer between 8 and 18 mN/m.

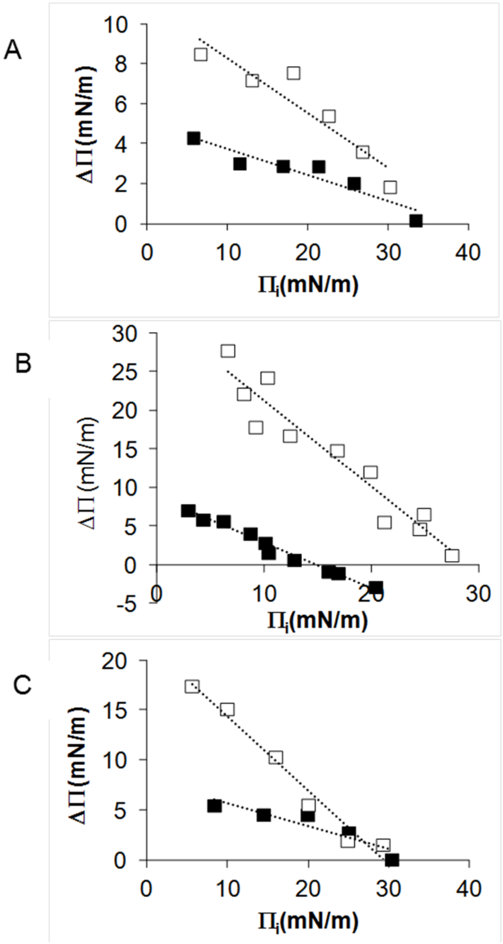

Figure S7. Surface pressure increase at equilibrium as a function of the initial surface pressure Pi) of the lipid monolayers induced by CitA (white squares) or CitO (black squares) adsorption. (**A**) PLPC, (**B**) Sitosterol, (**C**) PLPC/sitosterol (80/20) monolayer. Each point was obtained from an independent experiment.

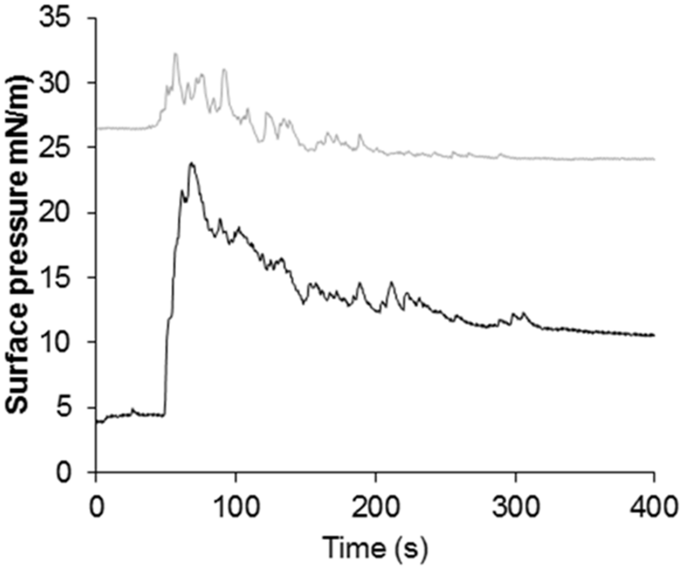

Figure S8. Adsorption of CitO into a β-sitosterol monolayer. Evolution of the surface pressure with time: in black at an initial surface pressure of 4 mM/m and in grey at an initial surface pressure of 26 mN/m.
